# Late onset hereditary sensory and autonomic neuropathy with cognitive impairment associated with Y163X prion mutation

**DOI:** 10.1007/s00415-014-7521-6

**Published:** 2014-10-07

**Authors:** Andreas C. Themistocleous, Robin Kennett, Masud Husain, Jacqueline Palace, Simon Mead, David L. H. Bennett

**Affiliations:** 1 Nuffield Department of Clinical Neurosciences, John Radcliffe Hospital, Level 6, West Wing, Oxford, OX3 9DU UK; 2 Medical Research Council (MRC) Prion Unit, The National Prion Clinic, University College London (UCL) Institute of Neurology, Queen Square House, Queen Square, London, WC1N 3BG UK

Dear Sirs,

A 69-year-old male presented with a 16-year history of numbness and paraesthesia of both feet and symptomatic postural hypotension. He reported episodes of painless thermal injuries to his limbs and lower urinary tract symptoms including reduced urinary flow, frequency, nocturnal incontinence and recurrent urinary tract infections. There was a long history of diarrhoea which preceded his sensory symptoms by 10 years and resulted in 20 kg of weight loss, and increasing memory difficulties with greatest difficulty in recalling recent events. His mother had suffered from symptomatic postural hypotension and foot ulceration developing in her sixth decade; she died in her seventh decade. It is not known whether she suffered from chronic diarrhoea or cognitive impairment. He has two brothers whom are unaffected.

Blood pressure was 156/92 lying and 79/53 standing. On neurological examination, cranial nerves were normal and there was mild wasting of the intrinsic foot muscles more marked on the right than the left. Tone and motor strength was normal. On sensory examination, pinprick was reduced to the mid-calf bilaterally, vibration sense was reduced to the knees, light touch to the ankles and joint position sense was reduced at the hallux. His ankle jerk reflexes were absent. His gait was hesitant. He was unable to perform tandem gait and Romberg’s sign was positive.

Neurophysiological studies are summarised in Table 1 and showed a length-dependant predominantly axonal motor and sensory peripheral neuropathy with subtle demyelinating features. Cognitive testing revealed impairment with major deficits, most prominently in memory and executive function. Table 2 summarises all remaining investigations. Of note is the absence of any evidence of systemic amyloid.

**Table 1:** Summary of neurophysiological studies

Motor studies	1997		2013	
	Right	Left	Right	Left
Nerve				
Tibial				
Latency (ms)	**5.8**	**5.4**		**6.5**
Amplitude (mV)	**0.8**	**1.1**		**0.05**
Conduction velocity m/s	**36**	**36**		
F Wave (max) ms	**65.5**	**Absent**		**Absent**
Peroneal				
Latency (ms): ankle				**5.45**
Amplitude (mV): ankle				**0.3**
Amplitude (mV): above knee				**0.3**
Conduction velocity (m/s)				**30.5**
F Wave (max) ms				**Absent**
Ulnar				
Latency (ms): wrist			3.30	
Amplitude (mV): wrist			11.7	
Amplitude (mV): above elbow			9.6	
Conduction velocity (m/s)			45.6	
F Wave (min) ms			33.15	
Median				
Latency (ms): wrist	4.3	3.90	3.75	
Amplitude (mV): wrist	18.8	16.0	14.9	
Amplitude (mV): elbow	17.3	14.8	7.1	
Conduction velocity (m/s)	52	48	43.3	
F Wave (min) ms			34.25	

Sensory studies	1997		2013	
	Right	Left	Right	Left
Nerve				
Sural				
Latency (peak) ms	**Absent**	**Absent**	**Absent**	**Absent**
Amplitude (peak–peak) µV	**Absent**	**Absent**	**Absent**	**Absent**
Radial				
Latency (onset) ms			1.90	
Latency (peak) ms	2.80	2.80	2.60	
Amplitude (peak–peak) µV	10.0	10.0	**0.26**	
Conduction velocity (onset) m/s			44.7	
Ulnar				
Latency (peak) ms	3.0	3.4	**Absent**	**Absent**
Amplitude (peak–peak) µV	**3.1**	**1.0**	**Absent**	**Absent**
Median				
Latency (peak) ms	4.10	4.10	**Absent**	**Absent**
Amplitude (peak–peak) µV	**2.20**	**2.10**	**Absent**	**Absent**

Electromyography	1997	2013
Muscle		
Right tibialis anterior	No spontaneous activity, Normal pattern of motor unit recruitment with motor units up to 2.5 mV	No abnormal spontaneous activity. Voluntary recruitment moderately severely reduced in density with an excess of slightly unstable irregular motor unit potentials: durations up to 20 ms, amplitude up to 10 mV
Right first dorsal interosseous	No spontaneous activity, Normal pattern of motor unit recruitment with motor units up to 2.5 mV	

The neurophysiological studies were consistent with a length-dependant predominantly axonal motor and sensory peripheral neuropathy. However, the slowing of the conduction velocities, prolonged F waves and conduction block of the median nerve suggest a possible demyelinating component. Sympathetic skin response (performed only in 2013) was absent in the right hand and left foot

Bold values indicate abnormal results

*ms* milliseconds, *mV* millivolt, *m/s* metres per second, *µV* microvolt

**Table 2:** Summary of relevant investigations

Blood tests
Hb 11 g/dl, WBC 5.34 × 10^9^/L, platelets 119 × 10^9^/L, MCV 101.5 fl, neutrophils 3.56 × 10^9^/L
Clotting screen normal
Urea 10.1 µmol/L, creatinine 151 µmol/L, eGFR 41 ml/min
Albumin 37 g/L
Liver function tests normal
IgA 2.2 g/L, IgG 9.8 g/L, IgM 1.2 g/L, no serum paraprotein detected
Serum free light chains: Kappa 26.8 mg/L, Lambda 20.9 mg/L. K/L ratio 1.28
Lipid profile normal
CRP <1 mg/L
NT- pro BNP 129pMol/L
Cardiac Troponin 0.015T ng/mL
Urine
Creatinine clearance 35.6 ml/min
24 h urinary protein loss 0.31 g
Bence Jones protein not detected
Genetic testing
PMP22 duplication/deletion negative
Transthyretin, SPTLC1, Rab7 sequencing negative
Amyloid deposition investigations
SAP Scan: no visceral organ uptake
DPD Scan: no cardiac uptake
Fat aspirate: no Congo red staining
Other tests
MRI brain: age appropriate cerebral atrophy
ECG: normal sinus rhythm
Echocardiogram: IVSd 1.1 cm. Mild TR. No evidence of cardiac dysfunction.

Genetic testing revealed a recently described Y163X truncation mutation of the gene encoding prion protein (*PRNP*) [1]. Prion protein is tethered to the cell membrane via a glycosylphosphatidylinositol anchor and the Y163X mutation results in a premature stop codon such that the truncated prion protein is no longer membrane anchored. The *PRNP* Y163X mutation, follows an autosomal dominant inheritance pattern, presents with adult onset chronic diarrhoea, followed by symptoms of a mixed, predominantly sensory and autonomic neuropathy and late onset cognitive decline and seizures. Pathologically prion protein is deposited throughout peripheral organs primarily the bowel and peripheral nerves, either in between nerve fibres [1] or as ragged deposits in the myelin [2] with significant fibre loss and no evidence of demyelination, and the cortex in the form of amyloid plaques, amyloid angiopathy, tauopathy and unique prion protein fragments. Pan-autonomic failure and peripheral neuropathy is probably due to abnormal prion deposition within the peripheral nervous system. Direct toxic effects of prion protein, deposited in bowel and urinary bladder, may contribute to severe diarrhoea and urinary symptoms. We have not studied adjacent markers on chromosome 20 to define whether there is a common ancestor with the larger pedigree [1].

A 2-bp deletion in codon 178 of *PRNP* has been described in Japanese kindred causing a familial sensory and autonomic neuropathy with associated cognitive impairment [2]. A different insertional mutation of *PRNP* resulting in a truncation at position 163 has also been associated with hereditary sensory and autonomic neuropathy [3].

The clinical presentation of prion protein systemic amyloidosis caused by the *PRNP* Y163X mutation is distinct from previously described forms of hereditary sensory autonomic neuropathy [4] with prominent and early bladder and bowel symptoms, late onset sensory/autonomic neuropathy followed by cognitive involvement. The duration of symptoms and early onset of bowel dysfunction would also be atypical for amyloid neuropathy.

Interesting features of this case include some subtle demyelinating features noted on nerve conduction. Occasional cases of amyloid polyneuropathy have also been reported to show demyelinating changes [5]. Mild conduction slowing could represent direct toxicity of the prion protein on Schwann cells or even prion protein haplo-insufficiency as axonal prion protein has been shown to be required for myelin maintenance in mouse models [6]. This case demonstrates the importance of *PRNP* analysis, which should be considered in the investigation of unexplained diarrhoea with a sensory neuropathy and is an important differential of familial amyloid polyneuropathy.
